# Targeting HIF2α Translation with Tempol in VHL-Deficient Clear Cell Renal Cell Carcinoma

**DOI:** 10.18632/oncotarget.561

**Published:** 2012-11-08

**Authors:** Carole Sourbier, Gaurav Srivastava, Manik C. Ghosh, Sanchari Ghosh, Youfeng Yang, Gopal Gupta, William DeGraff, Murali C. Krishna, James B. Mitchell, Tracey A. Rouault, W. Marston Linehan

**Affiliations:** ^1^ Urologic Oncology Branch, Center for Cancer Research, National Cancer Institute; ^2^ National Institute of Child Health and Human Development; ^3^ Radiation Biology Branch, Center for Cancer Research, National Cancer Institute, National Institutes of Health, Bethesda, MD

**Keywords:** HIF, Tempol, RCC, VHL, IRP1, iron metabolism

## Abstract

The tumor suppressor gene, *Von Hippel-Lindau (VHL)*, is frequently mutated in the most common form of kidney cancer, clear cell renal cell carcinoma (CCRCC). In hypoxic conditions, or when there is a *VHL* mutation, the hypoxia inducible factors, HIF1α and HIF2α, are stabilized and transcribe a panel of genes associated with cancer such as vascular endothelial growth factor receptor (VEGFR), platelet derived growth factor (PDGF), and glucose transporter 1 (GLUT1). Recent studies in clear cell kidney cancer have suggested that HIF2α, but not HIF1α, is the critical oncoprotein in the VHL pathway. Therefore, targeting HIF2α could provide a potential therapeutic approach for patients with advanced CCRCC. Since iron regulatory protein 1 (IRP1) is known to inhibit the translation of HIF2α, we investigated whether Tempol, a stable nitroxide that activates IRP1 towards IRE-binding, might have a therapeutic effect on a panel of human CCRCC cells expressing both HIF1α and HIF2α. We first evaluated the protein expression of HIF1α and HIF2α in 15 different clear cell renal carcinoma cell lines established from patient tumors in our laboratory. Tempol decreased the expression of HIF2α, and its downstream targets in all the cell lines of the panel. This effect was attributed to a dramatic increase of IRE-binding activity of IRP1. Several cell lines were found to have an increased IRP1 basal activity at 20% O_2_ compared to 5% O_2_, which may lower HIF2α expression in some of the cell lines in a VHL-independent manner. Taken together our data identify Tempol as an agent with potential therapeutic activity targeting expression of HIF2α in *VHL*-deficient clear cell kidney cancer and illustrate the importance of studying biochemical processes at relevant physiological O2 levels.

## INTRODUCTION

In the United States, an estimated 64,770 new cases of kidney cancers will be diagnosed in 2012 and about 13,570 people will die of this disease [1]. The most common type of kidney cancers, clear cell renal cell carcinoma (CCRCC), is associated with mutations of the *VHL* gene [2,3,4]. In normal tissues, the product of the *VHL* gene is associated with ubiquitination and degradation of the hypoxia inducible factor (HIF) through an oxygen-sensing mechanism [5,6]. In normoxia, HIF1α and HIF2α are hydroxylated by prolyl-hydroxylases in an iron-dependent manner. This post-translational modification allows recognition of HIF by the VHL complex and leads to its degradation by the proteasome [7,8]. However, in the absence of oxygen, or in the presence of a mutated *VHL* gene, HIF1α and HIF2α are stabilized and induce the expression of a panel of transcriptional target genes such as VEGF, PDGF, and GLUT1, supporting the metabolic shift that underlies CCRCC tumorigenicity [9]. Even in presence of oxygen, clear cell kidney cancer cells with *VHL* gene mutation display a “pseudo-hypoxic” phenotype. Although the degradation of both HIF1α and HIF2α are regulated by VHL [10,11], HIF2α has been thought to be the dominant oncoprotein in VHL-deficient CCRCC cells [12,13,14]. It was also recently suggested that HIF1α may function as a tumor suppressor gene in VHL-deficient clear cell kidney cancer [15]. However, despite the proposed critical role of HIF2α in VHL-deficient CCRCC tumorigenesis, only a few HIF2α inhibitors have been described [16,17].

A molecular link between HIF2α expression and iron availability has recently been reported [18]. HIF2α, but not HIF1α, has an iron-responsive element (IRE) in the 5' untranslated region (UTR) of its mRNA. Thus, when the cells are deficient in iron, the iron regulatory proteins (IRPs) repress the translation of HIF2α protein by binding to its 5'IRE. In addition, Ghosh et al. have recently shown that a stable nitroxide, Tempol (4-hydroxy-2,2,6,6-tetramethylpiperidine-N-oxyl) partially restored the phenotypes of IRP2 knockout mice by activating the IRE binding activity of IRP1 and thus partially compensating for the loss of IRP2 [19].

In this report, using a panel of VHL-deficient CCRCC cell lines that express increased either HIF1α or HIF2α or both, we investigated whether Tempol inhibits the translation of HIF2α by inducing the IRE binding activity of IRP1 and whether Tempol might be a potential targeted therapy for CCRCC.

## RESULTS

### HIF1α and HIF2α expression in CCRCC cell lines

**Figure 1:**
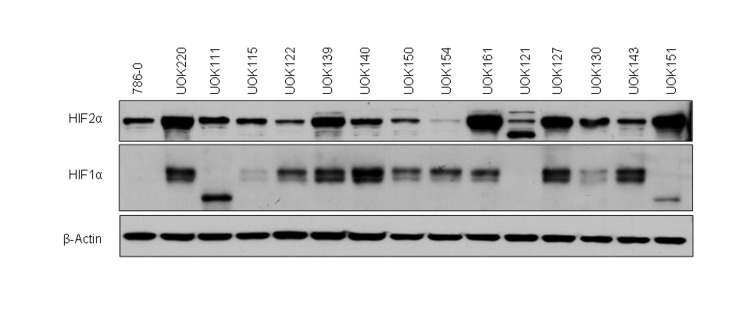
Characterization of the HIFα status of CCRCC cells Protein expression of HIF1α and HIF2α was assessed by immunoblotting. Fifteen different CCRCC cell lines (80% confluence) were lysed in TNSEV buffer, subjected to SDS-PAGE (20μg of whole cell lysates per well), transferred to PVDF membrane, and immunoblotted for HIF1α and HIF2α protein expression.

We first characterized the HIFα status of 15 RCC cell lines with mutated *VHL* gene (Figure 1 and Table 1). All of the cells expressed HIF2α, but some, including 786-0 [7], UOK111, UOK121 and UOK151 did not express HIF1α or expressed a shorter 75kDa splice isoform. Interestingly although expressed in UOK154, HIF2α protein levels were low relative to other *VHL*-deficient cells.

**Table 1:** CCRCC cell lines HIF status. Summary of the CCRCC cell lines and normal epithelial kidney cell line with their VHL mutation and their HIF status. NA: not available. Short: a 75kD isoform of HIF1α is expressed but not the full length

	Tissue Type	VHL Mutation	HIF1α	HIF2α
UOK111	Clear	missense T>A Trp117Arg	Short	Yes
UOK115	Clear	frameshift Del_TG Pro103	Yes	Yes
UOK121	Clear	A>G splice exon2/methylated	No	Yes
UOK122	Clear	nonsense C>T Arg113stop	Yes	Yes
UOK127	NA	2 hyper-methylated copies	Yes	Yes
UOK130	NA	T>C at NT. 671, Leu153Pro missense mt (exon 2)	Yes	Yes
UOK139	Clear	frameshift Del_A Thr 157	Yes	Yes
UOK140	Clear	frameshift Del_A Asp126	Yes	Yes
UOK143	Clear	A>G at 235 splice exon2	Yes	Yes
UOK150	Clear	Ins_C at 447 Tyr98; T>A at 448 Tyr98	Yes	Yes
UOK151	Clear	nonsense C>T Ser183stop	Short	Yes
UOK154	Clear	Ins_A Gln203	Yes	Yes
UOK161	Clear	C>T Gln164stop	Yes	Yes
UOK220	Clear	2 missense T>A Asn78Lys C>T Arg79Cys	Yes	Yes
786-0	Clear	1bp Del at NT. 523, frameshift at Gly104, exon 1	No	Yes
HK2	Normal	None	No	No

### Tempol decreases HIF2α protein expression and inhibits its downstream targets

**Figure 2:**
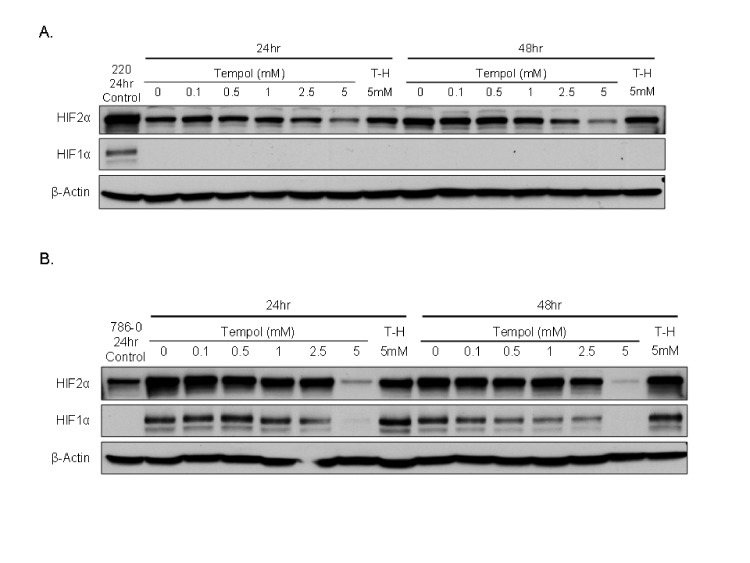
Tempol decreases HIFα expression A. To assess the effect of Tempol on HIFα expression in CCRCC cells, 786-0 cells were treated with several concentrations of Tempol for 24h or 48h before to be subjected to whole protein lyses, SDS-PAGE and immunoblotting for both HIFα isoforms. As previously reported 786-0 cells do not express HIF1α protein [7]. The CCRCC cell line UOK220 cell was used as a positive control for HIF1α and HIF2α antibodies. The reduced form of Tempol (hydroxy-Tempol, T-H) was also used as a negative control. B. A similar treatment regiment using Tempol was made using UOK220 cell line, a CCRCC cell line expressing both HIF1α and HIF2α. We used untreated lysates of 786-0 as a negative control for the HIF1α antibody and as a positive control for the HIF2α antibody. The reduced form of Tempol (hydroxy-Tempol, T-H) was also used as a negative control.

**Figure 3:**
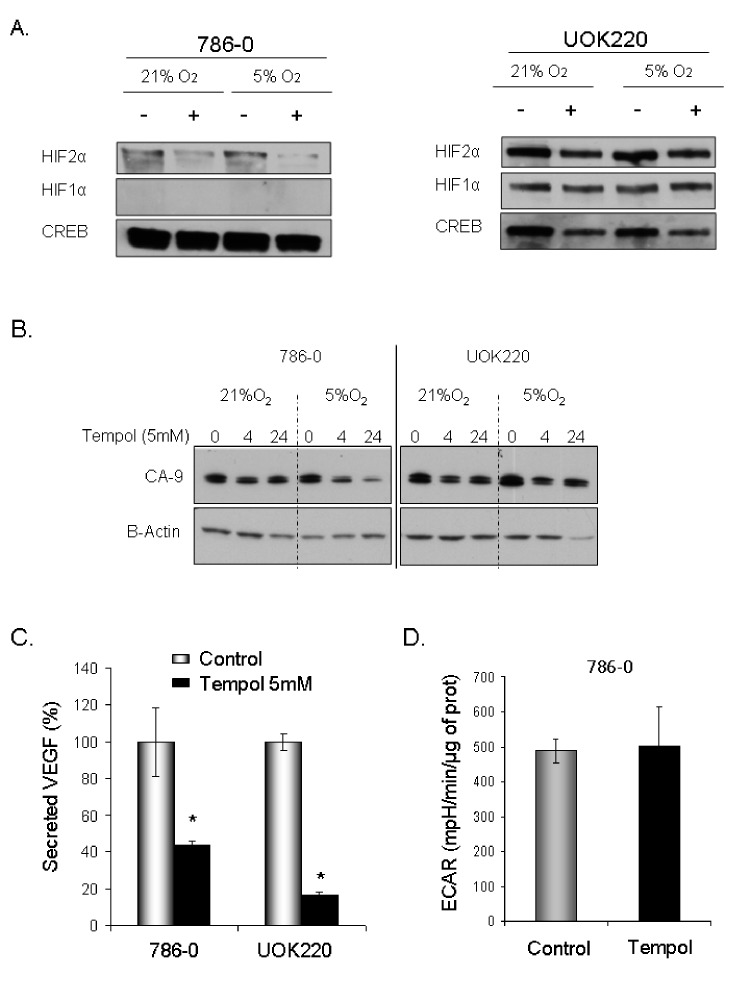
Tempol decreases HIF2α nuclear activity A. To assess the effect of Tempol on HIFα nuclear activities, expression of HIFα was assessed in 786-0 and UOK220 cells following Tempol treatment (5mM, 24h) and using 30μg nuclear lysates extracted at 21% or 5% O_2_. B. The effect of Tempol treatment (5mM, 24h) on the HIF2α transcriptional target, carbonic anhydrase 9 (CA-9), was visualized by immunoblotting using 20μg of whole cell extracts (786-0 and UOK220 cells). C. The effect of Tempol on another HIF2α transcriptional target VEGF was assessed by measuring the amount of VEGF secreted in the media of 786-0 and UOK220 cells after Tempol treatment (5mM, 24h). Secreted VEGF was quantified by ELISA using the MesoScale discovery (MSD) technology. D. The effect of Tempol on 786-0 cells’ metabolism was assessed using the Seahorse Bioscience technology.

**Figure 4:**
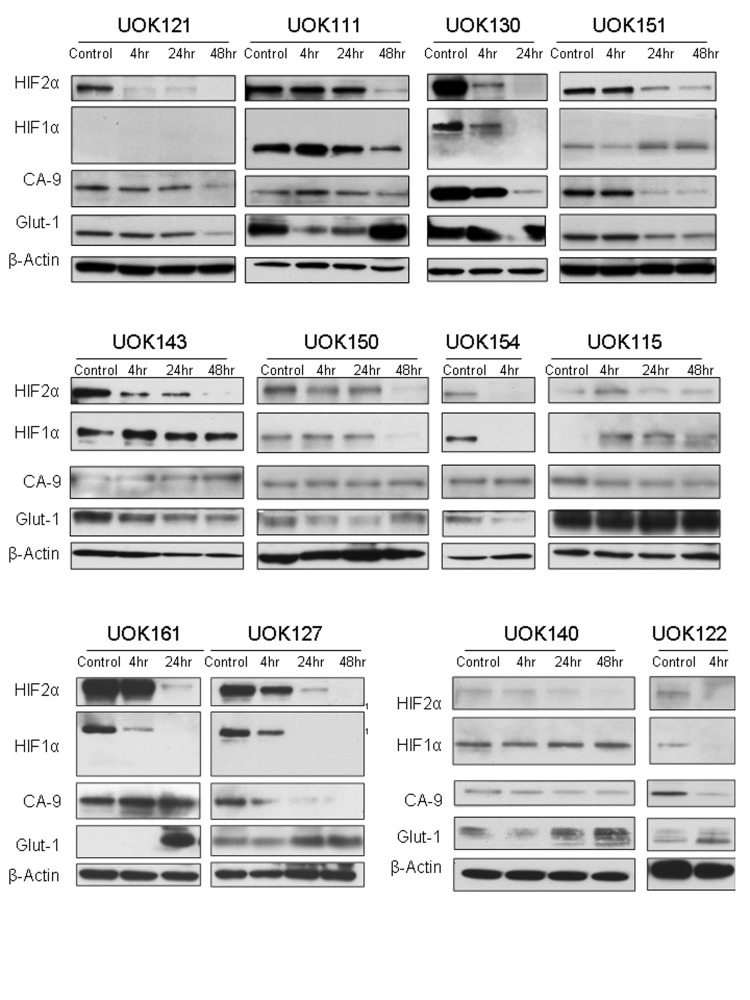
Tempol inhibits HIF2α in a panel of CCRCC cells Twelve CCRCC cell lines were treated with 5mM Tempol for 4h, 24h or 48h before to be subjected to whole protein lysis, SDS-PAGE and immunoblotting for both HIFα isoforms and CA-9. Twenty micrograms of whole cell extracts were used for loading the gels.

We then assessed the effect of Tempol on HIF1α and HIF2α protein levels. Tempol decreased HIF1α /HIF2α levels in 786-0 cell line, which expresses only HIF2α [7], and in UOK220 cell line, which expresses both HIFα isoforms (Figure 2). The reduced form of Tempol (Tempol-H), which was used as a negative control, had no effect on either HIF1α or HIF2α expression (Figure 2). To further investigate the effect of Tempol on HIFα activity and pathway, we assessed the level of nuclear HIF2α following Tempol treatment (5mM, 24h; Figure 3A, supplementary figure 1). Using 30μg of nuclear extracts and similar to that observed in the previous experiment, Tempol significantly decreased the level of nuclear HIF2α, suggesting that its transcriptional activity was also compromised. The effect of Tempol on nuclear HIF1α expression was, however, not consistent in all of the cell lines (Figure 3, supplementary figure 1). We then evaluated the expression of HIF2α downstream targets following Tempol's treatment. In 786-0 and UOK220 cells, both CA-9 expression and VEGF secretion, two known HIF2α targets and therapeutic targets for CCRCC [20,21], were found to be decreased after 24h of treatment with 5mM Tempol (Figure 3B-C). Also, since HIFα is known to play a role in regulating tumor cells metabolism, we asked whether Tempol might have a metabolic effect on 786-0 cells. As shown in Figure 3D and consistent with our observation that Tempol's effect on HIFα is mainly on HIF2α, Tempol (24h, 5mM) did not affect the extra-cellular acidification rate of 786-0 cells (ECAR), a surrogate for lactate secretion. This is consistent with other published works, such as the recent paper of Chiavarina *et al* which shows that HIF1α and not HIF2α is important for glycolysis in a breast cancer model [22]. Further, the effect of Tempol on HIF2α expression and on its downstream targets was highly reproducible since it was also observed in 12 other CCRCC cell lines displaying various HIF1α and HIF2α expression patterns (Figure 4 and Figure 1). Thus, our data suggest that Tempol is a potent HIF2α inhibitor, regardless of the HIF1α status of the CCRCC cell line studied.

### Tempol's effect on IRP1 activity correlates with HIF2α

**Figure 5:**
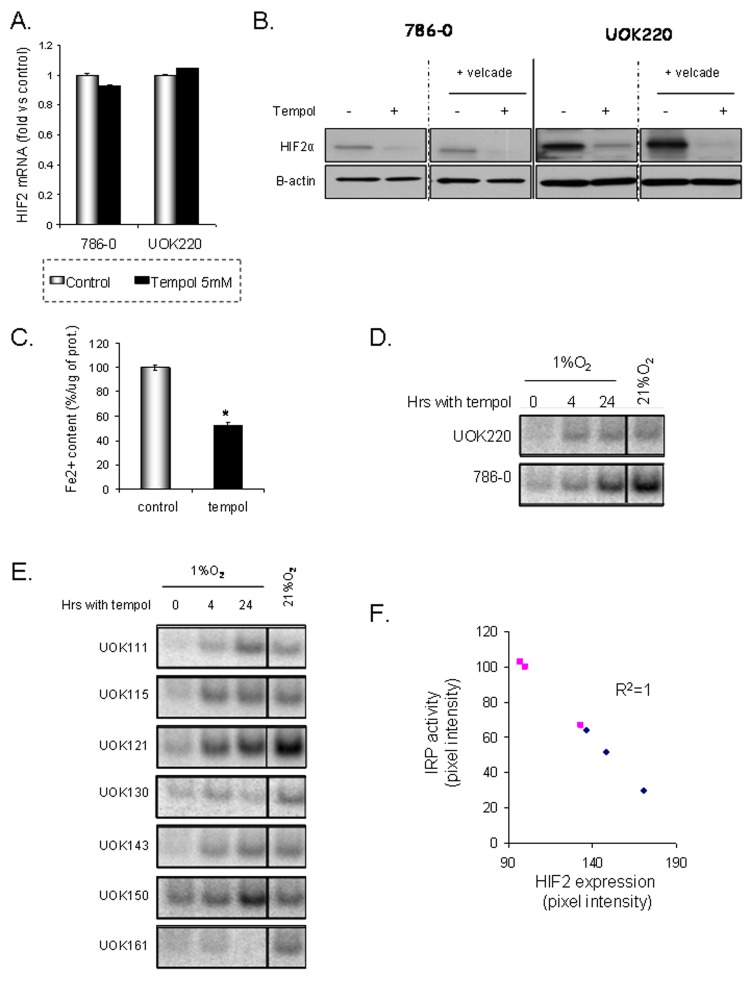
Tempol translationaly inhibits HIF2α A. The effect of Tempol (5mM, 24h) on HIF2α transcription was assessed by semi-quantitative RT-PCR in 786-0 and UOK220 cells. B. The effect of Tempol (5mM, 24h) on HIF2α protein degradation was assessed by treating the cells with the proteasome inhibitor velcade (1μM, 24h). HIF2α expression was then assessed by immunoblotting using 20μg of whole cell lysates. C. The amount of available ferrous iron (Fe^2+^) content was measured after Tempol treatment (5mM, 24h) in 786-0 cells. D-E. IRP1 activities of different CCRCC cell lines were measured at 1% and 21% O_2_ and following Tempol treatment (5mM, 24h). F. Correlation between HIF2α expression and IRP1 activity (R^2^ =1).

To further investigate Tempol's mechanism of action and how it decreases HIF2α expression, we first assessed whether Tempol might have an effect on HIF2α at a transcriptional level. We quantified HIF2α mRNA levels following Tempol treatment by semi-quantitative RT-PCR. As shown in Figure 5A, 24h of Tempol treatment did not significantly change the mRNA expression of HIF2α. Then,in order to assess whether Tempol might have an effect on HIF2α by inducing its degradation, we evaluated the effect of Tempol (5mM, 24h) on HIF2α expression following inhibition of the proteasome with velcade (1μM, 24h). As shown in figure 5B, using 20μg of whole cell lysates, Tempol still decreased HIF2α protein expression, suggesting that Tempol's effect on HIF2α happened at the translational level. Because HIF2α expression and iron availability have recently been linked [18] and that Ghosh *et al.* have shown that Tempol is able to activate the IRE binding activity of IRP1[19], we thus investigated Tempol's effect on iron availability and IRP1 activity. We measured the amount of Fe^2+^ in cells following Tempol treatment as the nitroxide agent Tempol is able to lower Fe^2+^[23]. As shown in Figure 5C, Tempol significantly decreased the amount of available Fe^2+^. In addition, Tempol increased IRP1 activity in a panel of CCRCC cell lines (Figure 5D-E), which inversely correlated with HIF2α expression (Figure 5F, R^2^=1). Taken together these data suggest that Tempol regulates HIF2α expression at the translational level by increasing the IRE binding activity of IRP1. Interestingly, the basal level of IRP1 activation was highly increased in most of the cell lines at 21% O_2_ (Table 2) [24], suggesting that the O_2_ environment of the cell culture *in vitro* also affects HIFα expression in *VHL-*deficient cells.

**Table 2:** Ratio of IRP1 activity status (21% to 1%) in CCRCC cell lines

Cells	Tissue Type	IRP1 activity at 20% O2 (fold compared to 1%O2)
UOK111	Clear	7.87
UOK130	NA	2.3
UOK143	Clear	3.26
UOK150	Clear	1.2
UOK161	Clear	2.06
UOK220	Clear	2.2
786-0	Clear	3.6

### Tempol is cytotoxic to CCRCC cells

**Figure 6:**
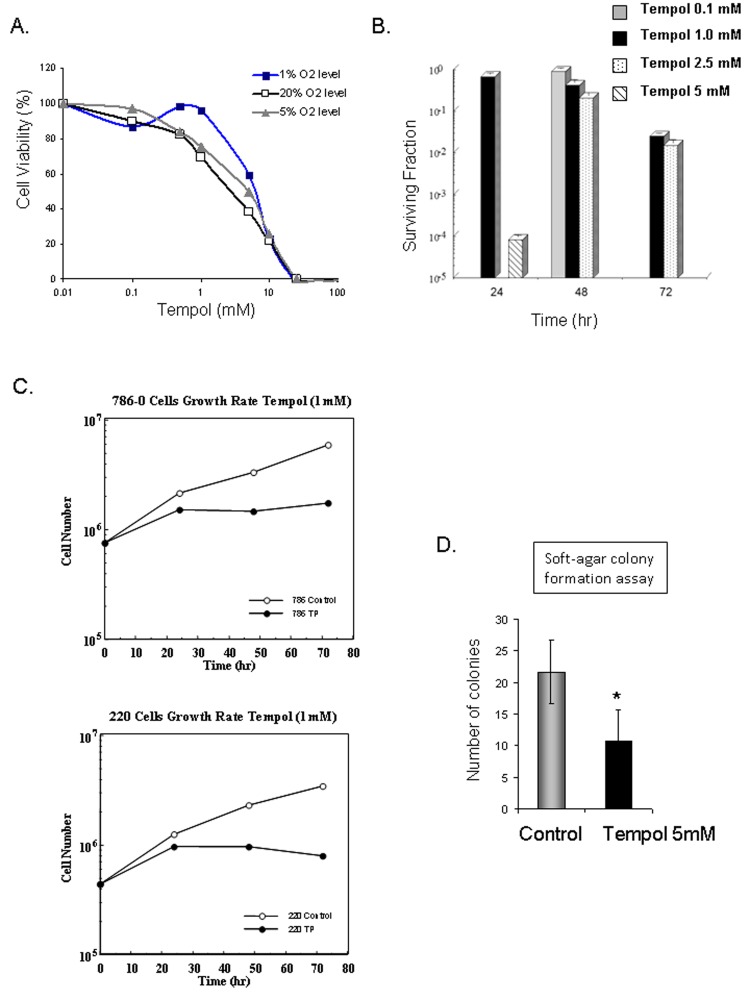
Anti-tumor effect of Tempol in CCRCC A. The cytotoxic effect of Tempol in 786-0 cells was assessed by MTT after 48h treatment at 1%, 5% and 20% O_2_. B. Cell viability following Tempol treatment was assessed by clonogenic assay in 786-0 cells. C. Effect of Tempol (1mM) on cell proliferation in 786-0 and UOK220 cells. D. The effect of Tempol (5mM) on the anchorage-independent growth ability of 786-0 cells was assessed by soft-agar colony formation assay.

Due to the observed effect of Tempol on HIF2α, CA9 and VEGF, all previously reported therapeutic targets for CCRCC tumors, we investigated whether Tempol might have an anti-tumor effect in CCRCC cells. Cell cytotoxicity assays demonstrated that 48h treatment with Tempol is cytotoxic to CCRCC cells *in vitro* (Figure 6A), that Tempol inhibits the formation of colonies of 786-0 cells (Figure 6B), and inhibits the proliferation of CCRCC cells 786-0 and UOK220 (Figure 6C). Also, Tempol treatment (5mM) decreased by 50% the ability of 786-0 cells to form anchorage-independent colonies, a marker of tumor transformation assessed by soft-agar colony formation assay (6D).

## DISCUSSION

Targeting HIF2α is of particular interest in cancer therapy since HIF2α is the transcription factor of numerous genes that have been involved not only in the development of CCRCC growth [20,21,25,26] but also in the development of other type of tumors [27,28]. However, to date, only few HIF2α inhibitors have been identified. In this report, we identified the nitroxide Tempol as a potent HIF2α inhibitor, with promising anti-tumor effects in a panel of CCRCC tumor cell lines.

Mutations occurring on the *VHL* gene are a critical event leading to CCRCC development in both sporadic and hereditary forms of kidney tumors. This lack of VHL function leads to the stabilization of both HIF1α and HIF2α proteins as well as to an increase in DNA damage [10,11,29]. Although the expression of both HIF1α and HIF2α is similarly regulated by VHL, their roles in tumor development, tumor metabolism, and response to therapy are sometimes contrasted [12,22,30,31]. Thus, although our goal was to identify a new HIF2α inhibitor, we characterized the expression status of both HIF1α and HIF2α in 15 VHL-deficient clear cell kidney cancer cell lines and assessed the effect of Tempol on both HIFα isoforms. Regardless of the differences in HIF1α and HIF2α expression between CCRCC cell lines, Tempol decreased the expression and function of HIF2α in all the cell lines. Surprisingly, Tempol also decreased the protein expression and activity of HIF1α in some of the CCRCC cell lines. As HIF1α does not have an IRE-binding domain, the reasons underlying Tempol's effect on HIF1α remain unknown, especially as it was inconsistent throughout the panel. Also, further work will be necessary to assess whether this effect on HIF1α might be beneficial or not for the survival of the tumors. Nevertheless, Tempol's effect on HIF2α was consistent in all the tested CCRCC cell lines regardless its effect on HIF1α.

The presence of an iron-responsive element in the 5'-UTR of HIF2α mRNA regulating HIF2α translation (but not HIF1α) links HIF2α and iron metabolism. This also opens new therapeutic strategies to selectively target HIF2α. When Ghosh and collaborators demonstrate that Tempol is able to activate the IRE binding activity of IRP1[19], we thus hypothesized that Tempol might also be able to inhibit HIF2α translation. In this report, we demonstrated that the decrease in expression of HIF2α following Tempol treatment significantly correlated with an increased activation of IRP1, reinforcing the link between IRP1 activity and HIF2α expression. Moreover, Tempol treatment decreased CCRCC cells viability, clonogenicity, proliferation, as well as anchorage-independent growth. Thus, by regulating the iron metabolism of CCRCC cells, Tempol may have a potential therapeutic value for cancers dependent on HIF2α for growth and survival, such as CCRCC tumors.

Interestingly, at 21%O_2_, most of the CCRCC cell lines (with the exception of UOK150) were found to have an artificially high level of IRP1 activity (Table2). This suggests that culturing CCRCC cells at 21%O_2_ may artificially lower HIF2α expression in a *VHL*-independent manner by oxidizing the [4Fe-4S] center in aconitase, and therefore, activating the IRE-binding form of IRP1. These findings highlight the importance of working in an *in vitro* condition that properly recreates the *in vivo* tumor environment [24].

Although significant progress in understanding the genetic basis of kidney cancer has been made over the past twenty years, there is still a need for the development of effective forms of therapy for patients with advanced kidney cancer. Since the identification of the *VHL* gene, seven novel agents that target the VHL pathway have been approved by the FDA. However, there are few complete responses to these agents and most patients develop resistance to therapy and develop progressive disease. Targeting HIF2α with an agent such as Tempol has the potential to inhibit the oncoprotein critical to clear cell kidney cancer carcinogenicity. The findings in this work may provide the foundation for the development of effective forms of therapy for this and other HIF2α-dependent cancers.

## METHODS

### Cell lines and cell culture

The UOK cell lines (UOK-111, 115, 121, 122, 127, 130, 139, 140, 143, 150, 151, 154, 161, and UOK220) were established in the Urologic Oncology Branch (National Cancer Institute, Bethesda, MD) [32]. 786-0 and HK-2 were obtained from ATCC. Cells were cultured in high glucose DMEM supplemented with 10% FBS. Cells were harvested or treated when they reached 70-80% confluence.

### Chemical agents

Tempol and hydroxyl-Tempol were purchased from Sigma-Aldrich (St Louis, MO).

### Immunoblotting

Ten to thirty micrograms of protein was loaded in 4-20% polyacrylamide gels (Biorad, Hercules, CA). After elelectropheresis, the proteins were transferred on PVDF membranes before being blocked with 5% fat-free milk for at least 1h. Primary antibodies were incubated over-night at 4°C. After serial washes with TBS-Tween, Horseradish peroxidase-linked secondary antibodies (Sigma-Aldrich) were incubated 1-2h before development with the ECL protein detection system (Pierce, Rockford, IL). Goat anti-HIF2α antibody was obtained from R&D Systems (Minneapolis, MN). Rabbit antibodies against β-actin, and carbonic anhydrase 9 (CA9) were from Cell Signaling Technology, Inc. (Danvers, MA). Mouse HIF1 antibody was from BD Transduction (San Jose, CA). All the antibodies were used at 1:1000 dilution.

### Cell cytotoxicity

Cell cytotoxicity was measured using the tetrazolium salt 3-(4,5-dimethylthiazol-2-yl)-2,5-diphenyltetrazolium bromide (MTT), as previously described [33]. Briefly, 5,000 cells were seeded into 96-well plate and treated as described in the results or figure legends. After 48 hrs, the MTT salt was added into the media (1:10) and incubated for 2 hrs at 37°C. During that time, metabolically active cells transformed the yellow MTT salt into purple formazan crystals. The media was then removed and the crystals were solubilized in 100 μl of DMSO. Data were read at 570nm. Cell survival was assessed by the clonogenic assay. Exponentially growing cells were exposed to Tempol (0.1 -5.0 mM) for 24-72 hr. Following treatment, cells were trypsinized, counted, plated, and incubated for 10-14 days. Colonies were fixed with methanol/acetic acid (3:1), stained with crystal violet, counted (colonies >50 cells), and survival was corrected for the plating efficiency of untreated controls.

### RT-PCR

Total RNA was extracted using RNeasy minin kit (Qiagen, Valencia, CA) and cDNA was prepared with High Capacity cDNA Reverse Transcription kit (Applied Biosystem). RT-PCR was performed using Platinum SYBR Green qPCR SuperMix-UDG (Invitrogen).

### RNA Mobility Shift Assays

Gel retardation assays were performed as follows: cell lysates were prepared in an anaerobic chamber in oxygen-depleted lysis buffer containing 10 mM Hepes (pH 7.2), 3 mM MgCl2, 40 mM KCl, 5% glycerol, 0.2% Nonidet P-40, 5 mM DTT, 1 mM the protease inhibitor AEBSF, 10 μg/ml Leupeptin and Complete EDTA-free protease inhibitor mixture (Roche Applied Science). Lysate (*x* μl) containing 10 μg of total protein was added to (12.5-*x*) μl of bandshift buffer containing 25 mM Tris-HCl (pH 7.5) and 40 mM KCl. The samples were incubated for 5 min at room temperature (RT) with 12.5 μl of a reaction mixture containing 20% glycerol, 0.2 units/μl Super RNAsine (Ambion), 0.6 μg/μl yeast t-RNA, 5 mM DTT, and 20 nM 32P-labeled IRE from human ferritin H chain gene in 25 mM Tris-HCl (pH 7.5) and 40 mM KCl. A measure of 20 μl of this reaction mixture was loaded into a 10% acrylamide/TBE gel, which was run at 200 V for 4 h, and then the gel was fixed, dried, and exposed for autoradiography.

### Determination of Fe2^+^

The content of ferrous ion was assessed using Iron Assay Kit (Biovision, Milpitas, CA) following the manufacturer instructions and without iron reducers. Fe^2+^ contents were measured at 593 nm using a microplate reader.

### Anchorage-independant colony formation assay

The ability of 786-0 cell line to form anchorage-independent colonies was assessed by soft-agar colony formation assay. Two layered agarose gels of 0.5% (bottom) or 0.4% (top) low melting agarase gels were pored into 60mm dishes. Two hundred thousands 786-0 cells were added to the top layer and cultured until colonies were formed (about 3 weeks). Colonies with a diameter of > 0.1 mm were then counted in 5 random high-power fields using a phase contrast microscope.

### Statistics

All values are expressed as mean ± Standard Error. All experiments were performed three times. Values were compared using the Student-Newman-Keul's test. P < 0.05 was considered significant.

## Supplementary Figures


