# Supplementary material for: Targeting HIF2α Translation with Tempol in VHL-Deficient Clear Cell Renal Cell Carcinoma
Source: Oncotarget. 2012 Nov 8;3(11):1472–82. doi: 10.18632/oncotarget.561 (PMC3717806; doi:10.18632/oncotarget.561)
Supplement: Supplementary file 1 [file oncotarget-03-1472-s001.pdf]

Targeting HIF2α Translation with Tempol in VHL-Deficient Clear Cell Renal Cell Carcinoma - Sourbier et al

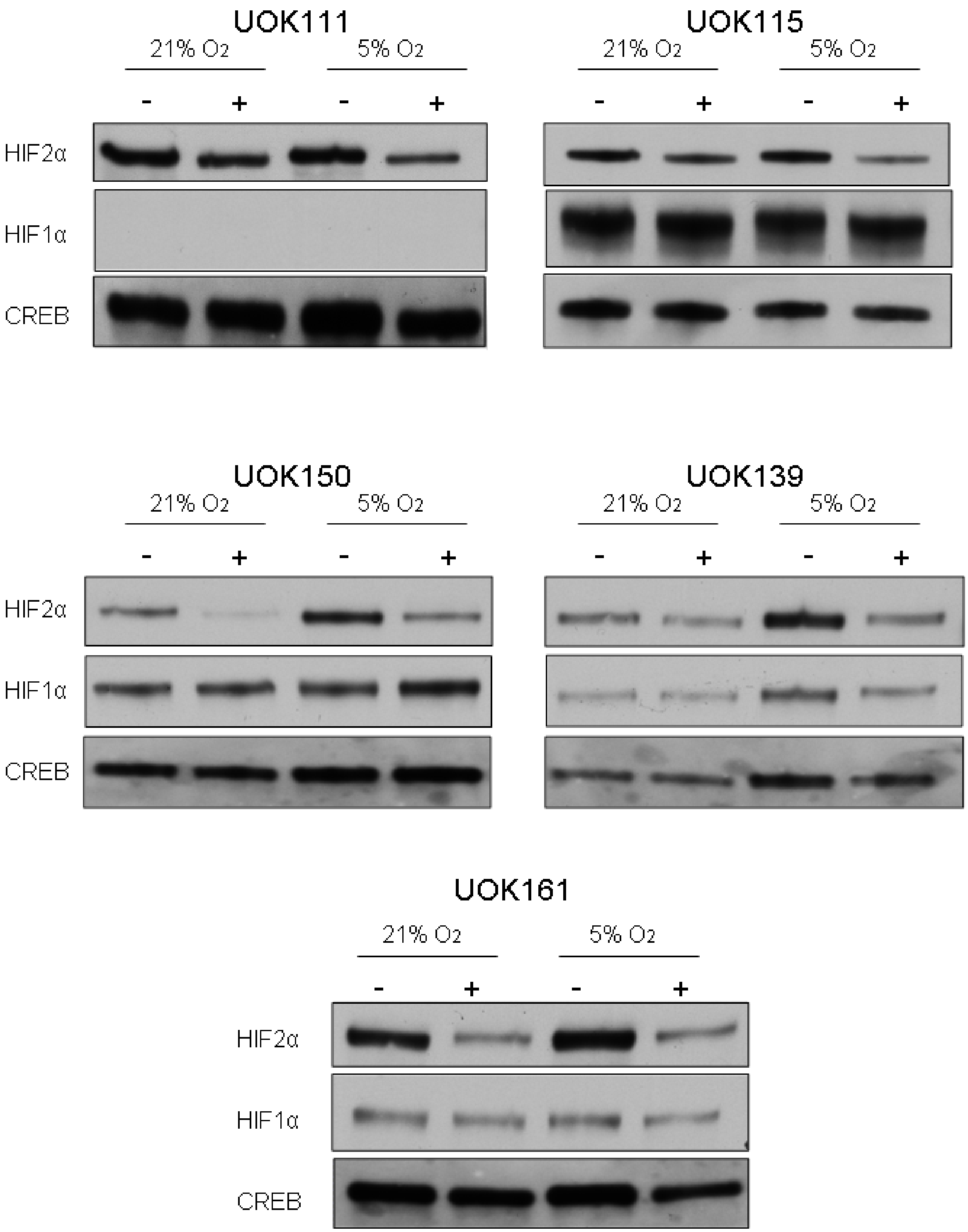

Supplementary figure 1: Tempol decreased nuclear HIF2 in other CCRCC cells. The effect of Tempol treatment (5mM, 24h) on the nuclear expression of HIF2α in a panel of CCRCC cell lines was assessed by immunoblotting using 30µg of nuclear extracts.
